# Emerging Evidence on *Tenebrio molitor* Immunity: A Focus on Gene Expression Involved in Microbial Infection for Host-Pathogen Interaction Studies

**DOI:** 10.3390/microorganisms10101983

**Published:** 2022-10-07

**Authors:** Giulio Petronio Petronio, Laura Pietrangelo, Marco Alfio Cutuli, Irene Magnifico, Noemi Venditti, Antonio Guarnieri, Getnet Atinafu Abate, Delenasaw Yewhalaw, Sergio Davinelli, Roberto Di Marco

**Affiliations:** 1 Department of Medicine and Health Science “V. Tiberio”, Università degli Studi del Molise, 8600 Campobasso, Italy; 2 Department of Biology, College of Natural Sciences, Debre Markos University, Debre Markos P.O. Box 269, Ethiopia; 3 School of Medical Laboratory Sciences, Faculty of Health Sciences, Jimma University, Jimma P.O. Box 307, Ethiopia; 4 Tropical and Infectious Diseases Research Center, Jimma University, Jimma P.O. Box 378, Ethiopia

**Keywords:** *Tenebrio molitor*, immunity, host-pathogen interaction, alternative insect model

## Abstract

In recent years, the scientific community’s interest in *T. molitor* as an insect model to investigate immunity and host-pathogen interactions has considerably increased. The reasons for this growing interest could be explained by the peculiar features of this beetle, which offers various advantages compared to other invertebrates models commonly used in laboratory studies. Thus, this review aimed at providing a broad view of the *T. molitor* immune system in light of the new scientific evidence on the developmental/tissue-specific gene expression studies related to microbial infection. In addition to the well-known cellular component and humoral response process, several studies investigating the factors associated with *T. molitor* immune response or deepening of those already known have been reported. However, various aspects remain still less understood, namely the possible crosstalk between the immune deficiency protein and Toll pathways and the role exerted by *T. molitor* apolipoprotein III in the expression of the antimicrobial peptides. Therefore, further research is required for *T. molitor* to be recommended as an alternative insect model for pathogen-host interaction and immunity studies.

## 1. Introduction

Vertebrate animal models are widely used for microbial pathogenicity studies, pharmacological testing, vaccine production, antibiotics effectiveness and safety evaluation, and pre-clinical screening studies [1]. Due to ethical issues, economically skilled labour, and time-consuming protocols, these models are increasingly giving way to new alternative strategies involving the use of invertebrates as new alternative animal models [2].

These new models are based on the principle of the 3Rs, introduced in 1959 by Willian Russell and Rex Burch in the book “*Principles of Humane Experimental Technique*” [3]. The acronym stands for “Refinement”, i.e., a better design of procedures performed on animals to minimise pain and/or stress; “Reduction” of the number of animals in the experiments, adopting alternative techniques to obtain reliable results; and “Replacement”, i.e., vertebrate substitution by non-sentient animals. 

In this scenario, because of vertebrates’ innate immune system similarity, insects as well as invertebrates have been increasingly chosen as an alternative model for host-pathogens interaction and immunity studies [4]. Although adaptive immunity is absent, insects exhibit a complex innate immune system with both cellular and humoral elements [5]. 

As regards the cellular responses, they are mediated by hemocytes, which play an essential defence role through phagocytosis, nodule formation, and encapsulation [6]. On the other hand, the innate humoral response is orchestrated by several processes that inhibit the pathogen’s proliferation and assist in its elimination, namely melanisation, hemolymph coagulation, synthesis of reactive compounds, and antimicrobial peptides (AMPs) [7,8]. 

It should be pointed out that insect immune system division into humoral and cellular responses is a narrative rather than a functional demand. Indeed, insect humoral response regulates many cellular mechanisms that depend on the activity of hemocytes [9]. In this scenario, different types of hemocytes that differ in morphology, molecular and antigenic markers, and function have been typified both in the larval and nymphal stages [10]. Therefore, hemopoiesis and hemocyte differentiation are closely related to immune system signalling pathways. For instance, hemocyte-mediated defence responses such as phagocytosis and encapsulation require the hemocytes’ host target recognition in order to trigger the activation of effector responses [6,11].

Compared to classical mammalian models, invertebrates breeding is less expensive and easy to maintain. In addition, the short lifecycle allows for large-scale experiments in a short period. Notably, the fruit fly *Drosophila melanogaster* [12], *Caenorhabditis elegans,* and the greater wax moth *Galleria mellonella* [13,14,15] belong to Diptera, Rhabditida, and Lepidoptera orders, respectively. These are the most widespread orders in the animal kingdom, with around 400,000 species that constitute almost 25% of all known animal life forms and occupy the majority of terrestrial environments [16].

The yellow mealworm *Tenebrio molitor* (Linnaeus, 1758) belongs to the order Coleoptera family Tenebrionidae. It is found worldwide; however, it has climatic preferences for temperate regions of the northern hemisphere, with an optimum growth temperature between 22 and 28 °C [17]. Mealworms are negatively phototropic and phototactic. Although photoperiod influences growth and development, the response tends to disappear under stable conditions when *T. molitor* becomes arrhythmic. [18]. *T. molitor* larval growth rate is optimal at ≥70% relative *humidity* (RH) with an *optimum* range between 60 and 75% RH. Similarly to the moth larva, it can be maintained at temperatures between 25 °C and 37 °C, which makes it a suitable model for host-human pathogen interaction [19]. Indeed, several studies on pathogenic fungi (*Candida albicans*, *Candida tropicalis, Cryptococcus neoformans*, and *Malassezia furfur*) along with Gram-negative (*Escherichia coli*) and Gram-positive (*Staphylococcus aureus* and *Listeria monocytogenes*) have been carried out [20]. 

Furthermore, the insect exhibits holometabolism (complete metamorphosis) after an incubation period of 7–8 days, and *larvae* will hatch from the eggs [21] with a four-stage lifecycle and a total developmental time of 80.0-83.7 days [22] (Table 1).

In addition, the larval phase presents a series of developmental stages or instars, which involves ecdysis at each stage [23,24,25]. 

*T. molitor* exhibits plasticity in a number of instars during larval development (from 9 to 20). According to Park et al., although the *larvae* at first instar were white, a gradual browning was observed from second instar onwards. Except for the colour change, the authors found no significant differences in the morphological characteristics of the *larvae*. During the progression of the larval stages, the body length gradually increased (from 0.34 ± 0.03 cm in 1st instar to 3.16 ± 0.13 cm in 17th instar), reaching a maximum at the 17th instar and decreasing thereafter. Indeed, about 69.69% of the total pupation was observed between the 15th and 17th instar, indicating that most of the analysed *larvae* had between 15 and 17 instars in their life cycle. Finally, the *larvae* were not significantly different in terms of incubation period or duration of the first instar. Between the second and 20th instar, the course of each instar was not uniform among the *larvae* [21].

*T. molitor* females lay around 500 eggs at a time, which hatch after 3–9 days. The larva lasts 1–8 months and is yellow light brown. The larva size is usually about 2.0–3.5 cm or more, and that of the adults is about 1 cm. It is omnivorous and can eat vegetables and animals; in commercial breeding, these specimens are fed mainly on bran or cereal flour (wheat, oats, and maise). 

Several abiotic and biotic parameters may influence the duration and number of larval stages, such as larvae nutritional status and growth temperature [18]. Pathogens may also cause *T. molitor* larvae to experience symptoms of malnutrition. Moreover, *T. molitor* gregarious nature increases its ability to resist pathogens [26]. Thus, the instars number increases in response to adverse conditions [27].

In this scenario, identifying the best rearing conditions is crucial for both the breeding yield in the food industry and laboratory protocol standardization. For instance, photoperiod influences the development and growth [28]. Under long-day conditions (14 h light, 10 h dark), shorter larval developmental times, longer pupal periods, and lowest eclosion rates were observed compared to other photoperiods [29]. 

Among biotic parameters that affect *T. molitor* development, the temperature must be taken into account. Although aspects of experimental conditions are often scarce, shorter larval developmental times (approximately 150 days) at 25 °C, compared to 30 °C (160–213 days) were recorded. However, [18], testing on the egg, larval, pupal, and adult survival at different temperatures, demonstrated an optimal temperature of 25 °C for high survival rates. On the other hand, survival rates decreased at 10 °C and 35 °C [17]. Additionally, it was discovered that larval age increased larval survival. [16]. The fastest larval development (about 111 days) was found at 30 °C while at 27.5 °C in 127 days [30]. The mealworms’ highest metabolic rate and daily wet mass increase were observed at 31 °C; the highest energy conversion efficiency was at 23.3 °C [31]. Finally, the highest survival and growth rates as well as the fastest development were assessed at 25 and 30 °C under continuous dark conditions [18].

Along with temperature, relative humidity (RH) has an impact on survival and mass rate. Under arid conditions (12% RH.) the egg gradually loses water and eventually dies from desiccation. While an RH of 52% and 75% along with extremes temperature (10 and 35 °C) do not significantly increase mortality [17]. At 30 °C, larval mass and length increase with humidity, those reared at 84% RH were 1.31 times longer and 1.96 times heavier compared to 43% RH [32].

Diet plays a crucial role in *T. molitor* life cycle, namely the development time, survival rate, number of instars, intensity and period of oviposition, and progeny production by increasing the number of eggs and decreasing adult mortality [28]. Compared to a carbohydrate-rich diet, a ratio of protein to carbohydrates 1:1 and a higher lipid content significantly improves lifespan and reproductive success [33,34]. Furthermore, protein supplementation increases larval development and rates survival rates from 84–88% to 88–92% and reduces pupal time from 191–227 days to 116–144 days at 28°C and 70%RH compared to low protein content regimen [35].

In addition to proteins, another important nutrient for larval development is vitamin B-complex. Indeed, *T. molitor* growth strictly depends on the presence of carnitine, thiamine, riboflavin, nicotinic acid, pyridoxine, or pantothenic acid, while the lack of biotin or pteroylglutamic acid considerably slows the growth rates [28].

Compared to other invertebrates models (Table 2), *T. molitor larvae* are relatively large, allowing the extraction of a considerable volume of hemolymph (5–10 µL/larva of the 12th instar) compared to the fruit fly *D. melanogaster*, (0.05–0.3 µL/larva of 3rd instar). Another advantage is that the inoculum can be administered by injection, allowing the direct pathogen insertion into the hemolymph [36,37]. On the other hand, in the *C. elegans* model, the pathogen is not parenterally administered but delivered together with the nematode in the bioassay. This results in a lack of accuracy regarding the microorganisms’ number internalised by the host [38]. 

Regarding genomic profiling, in 2014, Johnston et al. performed the transcriptome assembly and quantitative RNAseq analysis of gene coding for *T. molitor* immune system of insects infected with Gram-positive and Gram-negative bacteria. According to the authors, immune-responsive genes are regulated according to distinct temporal profiles during the infectious process. Those involved in antimicrobial peptide expression are persistently upregulated, while those responsible for constitutive defence responses have a transient upregulation. These findings made clearer the role of antimicrobial peptides in the containment of persistent infections [41].

More recently, on 27 January 2022, the chromosome-scale assembly of the yellow mealworm *T. molitor* genome was published by the National Centre for Biotechnology Information (NCBI) (GenBank GCA_907166875.3) by Eleftheriou et al. Authors combined RNA-seq data and available Coleoptera proteomes producing a high-quality genome with the completeness of 99.5% and predicting 21,435 genes with a median size of 1780 bp [42].

Although there are numerous studies concerning *T. molitor* immune system [20,43,44,45,46], recently several papers concerning the role of microbial infection on the expression of immune system genes have been published. Thus, this review aims at providing a broad view of the *T. molitor* immune system in light of the newest scientific evidence on the developmental/tissue-specific immunity gene expression studies related to microbial infection. All articles and findings are outlined in Supplementary Table S1.

## 2. *T. molitor* Gene Expression Studies Related to Microbial Infection

### 2.1. Nuclear Factor Kappa-Light-Chain-Enhancer of Activated B Cells (NF-Kb)

The most well-studied stimuli of insect NF-κB pathway are bacteria, fungi, and viruses. NF-κB is the nuclear transcription factor that plays a central role in the insect’s innate immunity, involving the expression of genes associated with the immune response to infections by pathogenic microorganisms. The two best-described routes to NF-κB activation are via Toll/Toll-like receptors (TLRs), or immune deficiency protein (IMD). Moreover, there are three types of NF-κB transcription factors identified in insects: Relish (Rel), Dorsal (Dor), and IkappaB kinase (IKK), which contribute to the regulation of Toll and IMD signalling pathways [47].

#### 2.1.1. Rel-Homology Domain (RHD)

**Figure 1:**
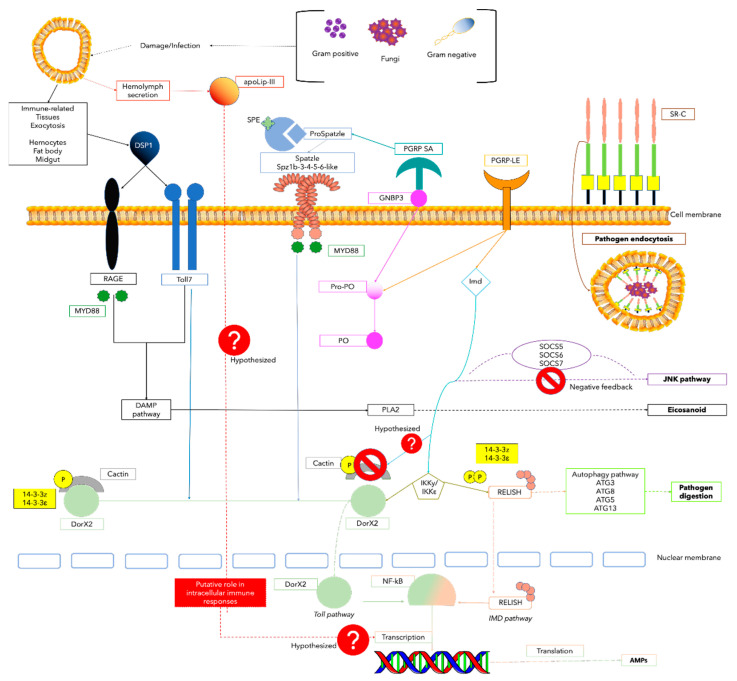
The main molecular mechanisms involved in the immune response in *T. molitor*. 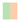 NF-kb: 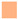 Rel-homology domain (Tm-Relish), 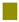 IkappaB kinase (Tm-yIKK and Tm-IKKε); 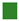 Myeloid differentiation factor 88 (Tm-MyD88) 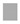 Cactin (Tm-Cactin); 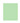 Dorsal protein (Tm-DorX2). 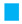 Immune deficiency death domain protein (Tm-IMD). 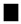 Damage-Associated Molecular Pattern: Dorsal switch protein 1 (Tm-DSP1) Tm-DSP1. 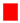 Apolipophorin III (Tm-apoLp-III). Immunity modulators: 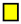 Tm-14-3-3 ζ and Tm-14-3-3ε; 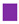 suppressors of cytokine signalling (Tm-SOCS5-6-7). 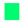 Autophagy (Tm- ATG3-8-5-13). 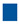 Toll receptors (Tm-Toll7). 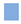 Spätzle (Tm-Spz1b-3-4-5-6-like). Peptidoglycan recognition proteins (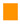 Tm- PGRP-LE, 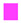 Tm-GNBP3, 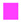 Tm-PGRP-SA). 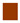 Scavenger receptors (Tm-SR-C).

The Rel-homology domain (RHD) protein, Rel, is a key NF-kB transcription factor of the IMD pathway that translocates into the nucleus upon bacteria detection and promotes the effector functions of AMPs (Figure 1) [48,49].

The first study to investigate its immunological function in *T. molitor,* the insect homolog Tm- (Tm-Rel) (Genbank MK863367), was conducted by Keshavarz et al. in 2020 [50]. To this end, in addition to protein structure studies and phylogenetic analyses, the authors analysed the mRNA expression profile both during *T. molitor* development in different tissues and *larvae* infected with *E. coli*, *S. aureus*, and *C. albicans*. Tm-Rel mRNA expression during the developmental stages was significantly higher in adults than in *larvae* or pupae and also in immune tissues such as hemocytes, gut, fat body, and MTs of both *T. molitor* late-instar *larvae* and 5-day-old adults. On the other hand, expression is not increased in the ovary or testis. Therefore, the increased expression of TmRelish in these tissues supported its role in inflammatory reactions. In late-instar *larvae* subjected to microbial infection, Tm-Rel mRNA was upregulated in the fat body, hemocytes, gut, and MTs after the *E. coli* challenge. In *S. aureus*-infected groups, the mRNA expression levels were lower in the fat body, hemocytes, and intestine than in the *E. coli*-infected ones. Furthermore, survival assays showed that Tm-Rel suppression caused an early and highly significant mortality rate in *E. coli*-infected *larvae* compared to cohorts infected with *S. aureus* and *C. albicans*. Finally, analysis of AMP gene expression in *larvae* with or without Tm-Rel knockdown revealed that expression levels were decreased with Tm-Rel silencing during infections. Tm-Rel knockdown had profound consequences on IMD pathway activation and AMP expression in both the gut and fat body in response to *E. coli*, demonstrating that these immunocompetent tissues are crucial elements of the immune response in terms of AMP production after *E. coli* infection. Furthermore, the negative regulation of certain AMPs by Tm-Rel in MTs, gut, and haemocytes in response to *C. albicans* infection had led the authors to hypothesise possible crosstalk between the Toll and IMD pathways [50].

Its role in *T. molitor larvae* upon infection with *L. monocytogenes*, via AMPs and autophagy genes (ATGs) regulation, was also investigated in another study by Keshavarz et al. in 2020. The authors examined Tm-Rel transcripts and AMPs and ATG genes induction in the fat body, haemocytes and gut of infected *larvae* silenced for Tm-Rel. The results demonstrated that both the expression of AMPs involved in the Gram-positive response (Tm-Tene1 and -4; Tm-Def1 and -2; Tm-Cole1 and -2, Tm-Att1a, -1b and -2; and Tm-Cec2) and ATGs (Tm-ATG1) was downregulated in the fat body and hemocytes of silenced *larvae* during *L. monocytogenes* infection. In contrast, the antifungal AMP genes, Tm-TLP1 and -2, were highly expressed in the fat body, and Tm-Def2 was significantly upregulated in the hemocytes and gut. Overall, this experimental evidence clarified the role Tm-Rel not only in the AMP genes regulation but also in the autophagy genes induction in response to L. monocytogenes infection. Furthermore, the different regulations exerted by Tm-Rel on AMPs in a tissue-specific manner confirmed the possible crosstalk between the Toll and IMD pathway [49].

#### 2.1.2. IkappaB Kinase (IKK)

In a 2020 published study, Ko et al. investigated the downstream regulatory components of the Toll signalling pathway and their function in the induction of AMPs (Figure 1) [51]. The authors elucidated the role of the γIKK complex in *T. molitor* (Genbank CAH1373157) in regulating AMP genes by RNAi screening studies upon microbial challenge. The study focused on the IKKγ/NEMO, the non-catalytic regulatory subunit of the IKK complex, which regulates the NF-kB signalling pathway. Experimental evidence on Tm-IKKγ mRNA expression in hemocytes, gut, and adipose tissues established its involvement in innate immunity. Furthermore, specimens silenced for this gene showed increased susceptibility to parenteral infection with the bacteria *E. coli*, *S. aureus*, and the fungus *C. albicans*. To understand which NF-kB component regulates the expression of *T. molitor* AMP genes, the authors assessed Tm-IKKγ role in the erythrocytes, gut, and fat body tissues of knockdown *larvae*. In infected *larvae* hemocytes and fat body, 10 AMP genes (Tm-Tene1, Tm-Tene2, Tm-Tene4, Tm-Def1, Tm-Def2, Tm-Cole1, Tm-Cole2, Tm-Atta1a, Tm-Atta1b and Tm-Atta2) and Tm-Rel were downregulated, whereas the expression of other NF-kB genes, including Tm-DorX1 and -X2, was only slightly downregulated. These findings suggested an IMD-dependent regulation by Tm-IKKγ in these tissues. In contrast, in the gut, the mRNA levels of two NF-kB genes (Tm-Rel and Tm-DorX2) were drastically downregulated, while Tm-IKK drastically increased the expression of Tm-DorX1 γ RNAi. These results demonstrated that Tm-IKKγ could activate both the Tm-Reldependent IMD pathway and the Tm-DorX2-dependent Toll pathway by positively and negatively regulating innate immune responses in *T. molitor* [51].

Thereafter in 2022, Ko et al. identified and functionally characterised an IKK homolog (Tm-IKKε) involved in immune signalling pathways regulation (Genbank MZ708789) [52]. The IKK is the NF-kB pathway’s central regulator against pathogen invasion in vertebrates and invertebrates. IKKbε and γ have critical roles in the Toll and IMD pathways; indeed, IKK family proteins act upstream of NF-kB factor by phosphorylating Rel and regulating the transcriptional activation of genes responsible for AMP transcription in the IMD pathway. Tissue-specific induction studies upon microbial challenge proved that mRNA expression was mainly induced by both *E. coli* and *S. aureus* parenteral infection leading to increased larval mortality in hemocytes. On the other hand, the negligible role of Tm-IKKε knockdown on NF-kB genes’ activation, namely Tm-Rel (NF-kB factor in IMD pathway), Tm-DorX1, and Tm-DorX2 (NF-kB regulators of the Toll signalling pathway), led to the hypothesis that Tm-IKKε was not required for AMP production in hemocytes. In the gut, the mRNAs of Tm-DorX1 and Tm-DorX2 were significantly downregulated in the silenced specimens, suggesting a role for Tm-IKKε in the regulation of transcriptional activation of AMP genes in the gut in response to systemic microorganism infection. In the fat body, RNAi silencing led AMP and NF-kB genes’ expression down-regulation in response to microbial challenges. These results encouraged the authors to consider Tm-IKKε as key regulator for the Toll and IMD pathways in the *T. molitor larvae* fat body by playing a critical function in AMP production and regulating the Toll (Tm-DorX1 and -X2) and IMD (Tm-Rel) pathways. In contrast, Tm-IKKε affects neither Tenecin 3 (AMP) gene expression nor larval mortality against fungal infection. Overall, this scientific evidence demonstrates the involvement of Tm-IKKε in antimicrobial innate immune responses in *T. molitor* [52].

#### 2.1.3. Myeloid Differentiation Factor 88 (MyD88)

MyD88 is known to play an essential role in the innate immune response of insects. The MyD88-dependent pathway mediates the antibacterial and antiviral responses in *D. melanogaster* and other invertebrates. MyD88 is an intracellular adaptor protein involved in the Toll signalling pathway and activation of the NF-κB transcription factor (Figure 1) [53].

Screening immune-related genes from *T. molitor* by Expressed Sequence Tag (EST) and RNAseq-based strategies, Patnaik et al. analysed the gene structure and biological function of Tm-MyD88 (Genbank HF935083) in this coleopteran. The gene consists of five exons and four introns, and its promoter includes binding sites for immune-related transcription factors. The authors also found that the expression of Tm-Myd88 was significantly upregulated in response to *S. aureus* and fungal β-1,3 glucan, but not the Gram-negative *E. coli*. In addition, Tm-MyD88 silencing attenuates resistance to *S. aureus* in *T. molitor*, suggesting that Tm-MyD88 is required for survival against infection due to the Gram-positive *S. aureus* [54].

#### 2.1.4. Cactin

Cactin was initially identified as an interactor of the Cactus factor in *D. melanogaster* IκB. It has been known to play a role in the control of embryonic polarity and regulation of the NF-κB signalling pathway (Figure 1) [55].

Jo et al. identified a Cactin homolog in *T. molitor*, Tm-Cactin (Genbank KY618833), and characterised its patterns of expression and its potential function in innate immunity [56]. Tm-Cactin mRNA was highly expressed in larval and prepubertal stages but at shallow levels in pupal and adult stages. Tissue expression results indicated that Tm-Cactin expression was detectable in all larval tissues tested but with high expression in the integument and MTs. As in *larvae*, Tm-Cactin expression in adults was relatively high in the integument and malpighian tubules compared to other tissues (intestine, fat body, hemocytes, ovaries, and testes). This evidence led the authors to assume that differences in Tm-Cactin expression between tissues and developmental stages may have to withstand hormonal regulation. Furthermore, expression analysis indicated an increase in Tm-Cactin in *larvae* after infection with *E. coli*, *S. aureus*, *C. albicans*, and *L. monocytogenes*. The expression of fourteen AMPs and the effect of RNAi knockdown after *E. coli* and *S. aureus* challenge showed that ten of the fourteen AMPs were induced by both *E. coli* and *S. aureus* infection, indicating that these AMPs are involved in the immune responses against these two bacteria. Seven of the nine AMPs that responded to bacterial infection (Tene1, Tene4, Def1, Def2, Cole1, Cole2 and Atta1b) showed significantly reduced expression after Tm-Cactin knockdown. In contrast, the expression increased for two of these (Tenecin2 and Attacin1a) following challenge with E. coli, but not with *S. aureus*. Given the involvement of the Toll pathway for Tenecin2 [57,58] and its high expression after Tm-Cactin knockdown, the authors hypothesised an activation mediated by other signalling pathways, such as the IMD pathway [56].

#### 2.1.5. Dorsal Protein (Dor)

Dor NF-κB transcription factors family member, is a critical downstream component of the Toll pathway that regulates AMPs expression by nucleus translocation and specific promoters binding (Figure 1) [59].

In 2019, Keshavarz et al. characterised the complete ORF of *T. molitor* Dor, Tm-DorX2 (Genbank MN056348), that housed a conserved Rel homologous domain (RHD) and an immunoglobulin-like domain, plexins and transcription factors (IPTs) [60]. The mRNA expression studies detected Tm-DorX2 at all developmental stages, with an increase in late larval and 2 days pupa with a maximum of 3 days in adults. Tm-DorX2 transcripts were highly expressed in adult MTs, fat body tissues, and larval MTs. To understand its involvement in *T. molitor* innate immunity, mRNA profiles following *E. coli*, *S. aureus*, and *C. albicans* challenges were assessed. The highest expression levels of Tm-DorX2 were observed 9 hours after infection in immune tissues (fat body, hemocytes, and intestine). *E. coli* exposure accelerated Tm-DorX2-Toll induction by increased transcript levels in the hemocytes and gut compared to *S. aureus* and *C. albicans* treated groups. Furthermore, AMPs gene expression levels in the fat body and gut of knockdown *larvae* after *E. coli* infection were more powerfully downregulated than those in hemocytes and MTs. Overall, the expression levels of AMPs in Tm-DorX2 knockdown *larvae* decreased significantly after bacterial and fungal challenges. These findings demonstrated that Tm-DorX2 acted as a negative regulator of these AMP genes in different tissues and a crosstalk between Toll and another immune signalling pathway, such as the IMD one. Lastly, the mortality rate of *T. molitor larvae* upon infection with *C. albicans* and *S. aureus* was higher than *E. coli*, suggesting that Tm-DorX2 was required to mount an innate immune response against *S. aureus* and *C. albicans* in the gut of the *larvae*, followed by a reaction in the fat body and hemocytes [60].

### 2.2. Immune Deficiency Death Domain Protein (IMD)

IMD is essential for humoral and epithelial IMD/NF-κB immune responses towards Gram-negative bacteria and viruses in insects. IMD is recruited together with the caspase DREDD and FADD in the immune signalling cascade after the mobilisation of peptidoglycan receptors (PGRPs). Activated IMD regulates the expression of AMPs, i.e., the immune response effectors against pathogens (Figure 1) [61,62].

For the first time in 2019, Jo et al. identified the IMD gene complete ORF in *T. molitor,* Tm-IMD (Genbank MK121950) [63]. Furthermore, functional characterisation studies allowed detection of cleavage and catalytic sites involved in the immune signalling cascade. The role of this protein in host survival during infections was investigated by expression studies and Tm-IMD silencing infected *larvae*. Tm-IMD expression was elevated in hemocytes and malpighian tubules of *T. molitor* late-instar *larvae* and adults. On the other hand, transcript knockdown significantly increased the mortality of *larvae* infected with *E. coli* and *C. albicans*. Moreover, the expression of nine AMPs of *T. molitor* (Tm-Tenecin1, Tm-Tenecin2, Tm-Tenecin4, Tm-Defensin2, Tm-Coleoptericin1, Tm-Coleoptericin2, Tm-Attacin1a, Tm-Attacin1b, and Tm-Attacin2) was significantly downregulated in Gram-negative infected knockdown *larvae*, while *C. albicans* challenge showed moderate expression of two AMPs (Tm-Defensin2 and Tm-Attacin2), suggesting possible involvement of Tm-IMD in AMP regulation against fungal infection. These results demonstrate the Tm-IMD’s involvement in the innate immune-mediated defence of the *T. molitor larvae* against Gram-negative and fungus infections through the induction of AMPs expression [63].

### 2.3. Damage-Associated Molecular Pattern (DAMP)

The first evidence for the presence of DAMP effector molecules in insects was reported by Mollah et al. for the lepidopteran *Spodoptera exigua* [64]. The authors demonstrated its role in PLA2 activation, leading to the production of the eicosanoids, well-known cellular and humoral immune response mediators. An effector of DAMP is the High-Mobility Group Box 1 (HMGB1), a nuclear protein ubiquitously expressed and highly conserved in almost all eukaryotic cells [65]. It is implicated in the activation of innate immune responses by interacting with recognition receptors such as receptors for advanced glycation (RAGE) and toll-like receptors (Figure 1) [66].

In a 2021 study, Mollah et al. identified the HMGB1 homolog in *T. molitor*, namely Dorsal switch protein 1 (Tm-DSP1) [67]. Although Tm-DSP1 (Genbank MW589636) functional domains analysis allowed the identification of two distinctive traits present in HMGB1, phylogenetic studies have shown that the protein is grouped with other insect DSP1s but separated from vertebrates and other animal HMGB1. Expression studies established that Tm--DSP1 was highly inducible in all tissues analysed, such as hemocytes, fat body, and midgut, where it was found at a high level compared to the other tissues. In order to verify the homologies between HMGB1and Tm-DSP1, its presence in the plasma of naive and immunocompromised *larvae* was assessed. Few Tm-DSP1 mRNA was detected in naive insect plasma. Whereas it was found in the immunocompromised ones, suggesting a possible excretion by cells capable of recognising an infection-associated signal in the same way as mammals [68]. The Tm-DSP1 RNAi knockdown demonstrated inhibition of both nodule formation and AMP expression in the treated *larvae*. Furthermore, although it is still unclear how Tm-DSP1 activates PLA2, a significant reduction in its activity was also observed in knockdown *larvae*. This enzyme catalyses the obligatory step for the biosynthesis of eicosanoids, mediators of cellular and humoral immune responses against various microbial pathogens such as bacteria, fungi, and viruses [69]. All this evidence allowed the authors to establish that the induction of Tm-DSP1 expression following bacterial challenge and its subsequent translocation could be associated with cellular and humoral immune responses by activating eicosanoid biosynthesis in *T. molitor.*

### 2.4. Apolipophorin III (apoLp-III)

ApoLp-III, together with apolipophorin I (apoLp-I) and apolipophorin II (apoLp-II), circulates in the hemolymph of many insect species and constitutes the protein component of lipoprotein particles essential for lipid transport in insect body. Due to its particular structure, in which five α-helices are associated with a hydrophobic interior and a hydrophilic exterior, apoLp-III shuttles in hemolymph between a lipid-free form and bound to lipophorin particles [70]. Despite this, several studies have also demonstrated that apoLp-III is involved in the innate host immunity of invertebrates, participating in different immune and stress responses [71,72]. In the insect immune reactions, apoLp-III acts as an antimicrobial factor or a molecule that detoxifies bacterial endotoxins. ApoLp-III can also influence phagocytosis and modulate the adhesive properties of hemocytes (Figure 1) [73].

Noh et al. cloned and characterised a novel Tm-apoLp-III (Genbank HG316496) homolog from *T. molitor*, which was organised into four exons interrupted by three introns. They also showed the presence of several immune-related transcription factor binding sites. Lower levels of apoLp-III transcript were detected in the larval and adult stages; vice versa, the highest levels were detected at late pupal stages. In addition, the transcript of Tm-apoLp-III was upregulated in the late stages of *L. monocytogenes* or *E. coli* challenge, suggesting that apoLp-III may play an essential role in innate immune responses against bacterial pathogens in *T. molitor* [74].

### 2.5. Immunity Modulators

Although it was believed that insects utilise Toll and IMD pathways for immune responses, recently, it was discovered that there are important modulators against infections. This section describes the regulation of these modulators in *T. molitor* exposed to microbial pathogens.

#### 2.5.1. 14-3-3 Proteins

The cellular signal transduction network is essential for mediating immune responses in insects. The 14-3-3 proteins constitute a group of conserved proteins in many eukaryotic organisms. Historically, this family of proteins has attracted much interest because they are involved in important cellular processes, including signal transduction, cell-cycle control, stress response, and immune signalling (Figure 1) [75]. In vertebrate and invertebrate models, the 14-3-3 proteins bind a wide array of cellular proteins, including kinases, phosphatases, receptors, and transcription factors [76]. At least two isoforms of 14-3-3, the ε and ζ, have been identified in insects such as *D. melanogaster* and *B. mori* [77,78]. These proteins have been shown to participate in both humoral and cellular immune responses in *D. melanogaster*. Still, it has also been suggested that 14-3-3 ζ plays a role in regulating developmental autophagy and diapause processes in insects [79,80].

Although the functional specificity of 14-3-3 proteins remains under investigation in *T. molitor*, Seong et al. demonstrated that Tm-14-3-3 ζ (Genbank KP099938) is essential in the host defence mechanisms against bacteria and fungi. In particular, the mRNA of Tm-14-3-3 ζ is higher expressed in the immune organs of the larval and adult stages of the insect and exhibited almost five-fold induction within 3 h post-infection of the *larvae* with *E. coli* and *Candida albicans*. Conversely, gene silencing of 14-3-3 ζ increased mortality of the *larvae* at 7 days post-infection, confirming its role in larval survival against *E. coli* and *C. albicans* [81].

A similar study demonstrated that the isoform Tm-14-3-3ε (Genbank KP099937) was required to control host viability under bacterial challenges. When infected with *E. coli*, the 4-3-3ε transcript shows a significant three-fold expression in the hemocyte of *T. molitor larvae*. Under silenced conditions, a reduction in AMP secretion appears to be responsible for loss in the capacity to kill bacteria, reducing the survival of the *larvae* upon *E. coli* infection [82].

#### 2.5.2. Suppressors of Cytokine Signalling (SOCS)

SOCS are intracellular proteins involved in various physiological processes in vertebrates and invertebrates. SOCS proteins have fundamental functions in development and homeostasis during immune responses. This protein family is induced by cytokines acting as negative feedback inhibitors. They control inflammatory signalling by regulating the extent and duration of the Janus Kinase-Signal Transducer, Activators of Transcription (JAK-STAT), and Epidermal Growth factor Receptor (EGFR) pathways (Figure 1) [83,84].

In *T. molitor*, Patnaik et al. have identified three SOCS genes (type I subfamily) [85]. The ORFs of Tm-SOCS5 (Genbank MK292064), Tm-SOCS6 (Genbank MK292065) and Tm-SOCS7 (Genbank MK292066) comprised 1389, 897, and 1458 nucleotides, coding for polypeptides of 462, 297 and 485 amino acids, respectively. Developmental expression analysis of the Tm-SOCS genes has revealed that Tm-SOCS5 and Tm-SOCS6 mRNA levels are highest at the egg stage. Instead, the Tm-SOCS7 mRNA was higher in the larval, pupal, and adult stages than in the egg stage. The expression of tissue-specific mRNAs in *larvae* demonstrated that Tm-SOCS5 and Tm-SOCS6 levels were higher in hemocytes than those observed in other tissues, whereas Tm-SOCS7 levels were higher in MTs. However, mRNA levels of the Tm-SOCS5, Tm-SOCS6 and Tm-SOCS7 transcripts were elevated in the adult ovaries. Therefore, the authors hypothesised that Tm-SOCS proteins could be an active factor in embryogenesis. Furthermore, upregulation of Tm-SOCS transcripts was observed in the hemocytes, fat body, and gut tissues of *T. molitor* after microbial infection suggesting that they are also crucial in immune response [85].

### 2.6. Autophagy

Autophagy is an evolutionarily conserved catabolic mechanism involved in many physiological processes, including cell survival, cell death, and immune response. The autophagic process refers to a group of intracellular degradation pathways that sequester a portion of the cytoplasm and/or organelles, thereby forming a double-membrane vesicle called the autophagosome. Subsequently, the intracellular material is delivered to the lysosome, the site of degradation of obsolete cellular material [86]. The formation of the autophagosome is a tightly regulated mechanism involving the expression of a series of proteins encoded by ATG genes. The ATG genes have been identified in many eukaryotes, including insects. ATG homologs have been characterised in *D. melanogaster* [87], *B. mori* [88], and *G. mellonella* [89]. Currently, more than 35 ATG proteins have been characterised in the formation of autophagosomes during the autophagic process. Although autophagy in insects has been investigated under conditions of nutrient depletion and during the developmental processes, its role in the innate immunity of insects has recently received considerable attention. Moreover, several studies have demonstrated that autophagy is essential for capturing and degrading bacteria, viruses, and parasites. In *D. melanogaster* the downregulation of ATG genes, such as ATG1-2-4-6-7-8-9 led to an increase in viral titer with time has been shown (Figure 1) [90].

Recently, genetic and functional evidence suggests that ATG genes are of pivotal importance in the autophagic process of *T. molitor*. The transcriptional profiles of Tm-ATG3 (Genbank KF670693) and Tm-ATG5 (Genbank KF670694) by RNA sequencing of *T molitor* indicated that these genes are ubiquitously and constitutively expressed, suggesting a role in the development and innate immunity. Moreover, the depletion of ATG3 and ATG5 by RNA interference demonstrated a crucial role of these genes in autophagy induction and survival ability of *T. molitor larvae* against an intracellular pathogen such as *L. monocytogenes* [91].

Similarly, Lee et al. revealed that Tm-ATG13 (Genbank KJ778621) is essential in initiating autophagy against bacterial pathogens such as *S. aureus* and *E. coli*. Gene expression analysis revealed that ATG13 is expressed ubiquitously during all developmental stages of *T. molitor,* and its silencing showed reduced survivability in response to bacterial infection [92].

In another study, ATG8 (Genbank KM676434) was required for autophagic clearance of *L. monocytogenes* in *T. molitor* has been identified. Loss of function of ATG8 by RNAi increased the mortality rates of *T. molitor larvae* against *L. monocytogenes*, suggesting that this gene plays a role in regulating autophagy-based clearance of *L. monocytogenes* in *T. molitor* [93].

Additionally, in insects, ATG6 plays a pivotal role in autophagosome formation and autolysosome maturation. The molecular function of Tm-ATG6 (Genbank MN259540) in *T. molitor* has been studied by Edosa et al. using qRT-PCR and RNAi-silencing approach. Tissue expression patterns of ATG6 were analysed in *T. molitor larvae* by infecting the beetle with *E. coli*, *S. aureus*, *L. monocytogenes*, and *C. albicans*. The results revealed that ATG6 is highly expressed in all tissues exclusively infected with the gram-positive bacteria *L. monocytogenes*. Moreover, the knockdown of the ATG6 transcripts reduced the larval survival rate against *L. monocytogenes*, indicating that ATG6 is directly involved in counteracting the infection of this pathogen in *T. molitor* [94].

### 2.7. Toll Receptors

Toll and Toll-like receptors (TLR) in insects and mammals play an important role in innate immunity. In response to microbial infections, insects mount a multifaceted immune response involving defence mechanisms mediated by the Toll signalling pathway, especially against Gram-positive bacteria and fungi (Figure 1).

While it is known that the Toll-7 receptor in *D. melanogaster* plays a critical role in antiviral autophagy [95], its involvement in antibacterial and antifungal immunity in *T. molitor* was elucidated by Park et al. in 2019 [96]. The authors demonstrated that Tm-Toll-7 (Genbank MK234903) expression was significantly induced in *larvae* 6 h after infection with *E. coli* and *S. aureus* and 9 h after infection with *C. albicans*. Furthermore, Tm-Toll-7 silencing in infected *larvae* made them more susceptible to *E. coli* but not to *S. aureus* and *C. albicans* suppressing a selection of AMP genes (Tm-Defensin-1, Tm-Defensin-2, Tm-Coleoptericin-1, and Tm-Attacin-2) belonging to AMP families with antibacterial activity against Gram-negatives [97]. Finally, the inhibition of Tm-attacin-2 expression in knockdown *larvae*, accompanied by structural and phylogenetic studies, led the authors to hypothesise an analogy between Tm-Toll-7 and 18W, a mediator of nuclear translocation necessary for the activation of AMP gene expression. However, unlike 18W, Tm-Toll-7 could be activated by Gram-negative bacteria [96].

### 2.8. Spätzle (Spz)

The extracellular proteins Spz play a crucial role in insect innate immunity by acting as a downstream ligand for Toll receptors. Throughout the infection, the PGRP and GNBP receptors recognise the meso-diaminopimelic acid (DAP)-type peptidoglycan (PGN) of Gram-negative bacteria and the lysine-type peptidoglycan of Gram-positive. After recognition, a proteolytic cascade activation that involves modular serine protease (MSP) allows the generation of active Spz by a cleaving process mediated by the Spz enzyme activating enzyme (SAE) and Spz-processing enzyme (SPE). The active Spz binding to the Toll receptor induces the expression of AMPs as a response to the invading pathogens (Figure 1) [58,98].

Regarding *T. molitor* Spz, Edosa et al. identified and functionally characterised a novel Spz receptor Tm-Spz6 (Genbank CAH1365756) [99]. Furthermore, its role in bacterial and fungal infections has been investigated by stage- and tissue-specific expression studies. Tm-Spz6 mRNA was highly expressed in prepupal and pupal stages, while lower expression levels in larval and adult. Therefore, it was hypothesised that Tm-Spz6 expression is regulated by developmental hormones. Tissue-specific expression studies demonstrated that although Tm-Spz6 was expressed in all larval and adult tissues, the highest expression was found in late *larvae* haematocytes. Moreover, in *larvae* inoculated with *E. coli*, *S. aureus* or *C. albicans,* Tm-Spz6 expression was highly induced in the three immune-related tissues (hemocytes, fat body, and intestine). Finally, The RNAi knockdown approach demonstrated the Tm-Spz6 involvement in microbial defence response. Silenced *T. molitor larvae* had significant susceptibility to *E. coli* and *S. aureus* infections together with suppressed expression of several AMP-encoding genes with both gram-negative (Tene-2, Cec-2, Tene-4, and Cole-1) and gram-positive bacteria activity (Tene-3, Def-1, Def-2, Cec-2, and Tene-1) [99].

After that, in 2020 the same authors reported the response of biosurfactants (bacitracin, fengycin, iturin A, and rhamnolipids) in modulating several *T.molitor* Spätzle, namely Spz3 (Genbank CAH1376064), Spz4 (Genbank MT075617), Spz6 (Genbank CAH1365756), and Spz-like (Genbank MZ708792) along with AMP expression [100]. In *larvae* inoculated with biosurfactants, the expression of AMPs through activation of Tm-Spz genes in hemocytes was observed. Furthermore, the biosurfactants tested significantly increased the survival of *T. molitor larvae* against *E. coli* after 24 h immune stimulation. Moreover, the biosurfactants’ effect on Spz-silenced *larvae* was investigated to confirm the protein involvement in the immune response induced by biosurfactants. Tm-Spz knockdown *larvae* were more susceptible to *E. coli* infection despite immuno-stimulation with biosurfactants compared to the control group *larvae* [100].

Another Spz isoform, Tm-Spz 1b (Genbank MZ708791), was identified and functionally characterised by Bae et al. in 2021 [101]. Developmental and tissue-specific expression profiles established that Tm-Spz1b was mainly expressed in adult hemocytes and the late larval stage. To understand the role of Tm-Spz1b in *T. molitor* innate immunity, temporal expression patterns of Tm-Spz1b against microbial challenges were investigated in hemocytes, fat bodies, and the gut. The mRNA expression of Tm-Spz1b was highly induced in the haemocytes 6 hours after microbial infection. However, higher mortality was observed in Tm-Spz1b knockdown *larvae* infected with *E. coli* but not *S. aureus* and *C. albicans*. Finally, Tm-Spz1b in AMPs expression and Toll/IMD signalling cascade was also investigated. AMP-coding genes of RNAi *larvae* hemocytes and fat were downregulated in response to the *E. coli* challenge and Tm-DorX2 transcripts. Thus, these results suggested that Tm-Spz1b was required to confer antibacterial defence against Gram-negative bacteria but not against Gram-positive and fungal infections [101].

Furthermore, Tm-Spz5 (Genbank MW916536) was characterised by Kojour et al. in 2021 [102]. mRNA expression studies at different developmental stages and in various tissues confirmed its expression at all developmental stages. However, the highest levels were observed in embryos and pupae, while lower ones were found in the late larval stage. Following bacterial and fungal *larvae* infection, Tm-Spz5 expression was significantly upregulated in a tissue-specific and time-dependent manner. In Malpighi tubes (MTs), significant upregulation was observed in response to *C. albicans* and *E. coli* at 3 hours after injection and *S. aureus* at 9 hours after infection.

Nevertheless, Tm-Spz5 knockdown *larvae* exhibited significant survival reductions when infected with *E. coli* and *S. aureus*, while the *C. albicans* infected showed similar survival rates compared to the control. AMPs expression data supported these findings. Indeed, Gram-negative and positive AMPs, such as Tenecins, Thaumatin-like-proteins, Attacins, Cecropin-2, and Coleoptericins, were significantly downregulated in MTs knockdown *larvae* infected with *E. coli* and *S. aureus*. On the other hand, the AMPs expression was comparable to control in *C. albicans* infection groups [102].

Finally, Jang et al. described a Tm-Spz-like (Genbank MZ708792) [103]. Tm-Spz-like ORF region had a length of 885 encodings for a 294 amino acid polypeptide. The protein comprises a cystine knot in the C-terminus containing conserved cysteine residues that can potentially bind to the Toll receptor. Tm-Spz-like mRNA expression was found in all developmental stages and tissues, with the highest expression in eggs and young larval stages. The effect of *E. coli*, *S. aureus,* and *C. albicans* challenge on Tm-Spz-like expression at different times in immune-related tissues, and the whole body was examined. Although it was expressed in all tissues and the whole body, the highest expression was observed in the intestine, fat bodies, and MTs 24 hours after microbial infection. Furthermore, in agreement with previous studies [99,100], Tm-Spz-like knockdown *larvae* exposed to *E. coli* exhibited significantly lower viability. These findings endorsed the downregulation of 11 encoding Gram-negative AMP genes (out of 15 genes tested). Additionally, the transcription factors involved in the Toll (Tm-DorX1 and X2) and IMD (Tm-Rel) pathways were downregulated in Tm-Spz-like-silenced *larvae* infected with *E. coli* [103].

### 2.9. Peptidoglycan Recognition Proteins (PGRPs)

PGRPs are evolutionarily conserved molecules belonging to the recognition receptor (PRR) family, which includes toll-like receptors in mammals and β-1,3-glucan recognition proteins (βGRPs) in insects and other invertebrates [104]. PGRPs play a key role in the host’s innate immune response, acting as sensors for bacterial peptidoglycan recognition and following induction of AMPs [105]. Insect PGRPs were first characterised in *B. mori* and *Trichoplusians* [106,107] but have not been found in plants or nematodes so far (Figure 1) [105].

PGRP-LE selectively binds the diaminopimelic acid (DAP) by activating the IMD and pro-phenoloxidase (proPO) pathways. Although most PGRPs act as extracellular microbial sensors, PGRP-LE has also been shown to play a role in intracellular bacterial recognition in *D. melanogaster* [108]. Tindwa et al. partially characterised a *T. molitor* homolog of -PGRP-LE (Genbank HF935084) and evaluated its role in survival against *L. monocytogenes* infection by RNAi knockdown. Although temporal expression patterns of Tm-PGRP-LE had demonstrated the constitutive gene expression at all developmental stages, RNAi *larvae* did not show a significant alteration of AMPs expression with or without infection. Nevertheless, Tm-PGRP-Le knockdown increased *T. molitor larvae* susceptibility to *L. monocytogenes* infection [109].

A study conducted by Keshavarz et al. in 2020 enlightened the role of Tm-PGRP-LE in gut AMPs production as a response to infections [110]. Tissue gene expression analysis demonstrated a high expression level in *larvae* and 5-day-old adults without microbial infections. On the other hand, due to *E. coli* and *C. albicans* challenge, TmPGRP-LE mRNA levels were markedly upregulated in both the fat body and gut. Knockout data supported these findings. Indeed, *T. molitor* was more susceptible to *E. coli* challenge and, to a lesser extent, *S. aureus* and *C. albicans*. The reduction of TmPGRP-LE had driven in downregulation of eight AMP genes in *E. coli, S. aureus*, and *C. albicans* infected insects along with remarkable effects on Tm-Rel, Tm-DorX1-X2 expression level in the fat body and gut. Taken together, all this evidence endorses the role of Tm-PGRP-LE as an important gut microbial sensor that turns on AMPs via IMD activation in response to *E. coli*; conversely, its role in AMPs synthesis was irrelevant in the hemocytes and fat body [110].

GNBP3 has a strong affinity for the β-1,3-glucan, a fungal cell wall component, which can activate the prophenoloxidase (proPO) cascade and induce the Toll signalling pathway. By RT-PCR and RNAi silencing, Yang et al. monitored the response of five key genes (Tm-GNBP3, Tm-MyD88, and Tenecin 1, 2, and 3) associated with the Tm-Toll pathway against the fungus *Beauveria bassiana* JEF-007. They demonstrated that Tm-GNBP3 (Genbank AB10884) was essential for inducing the gene expression of antifungal Tenecin 1 against infection with *B. bassiana* JEF-007 [111]

Together with GNBP, PGRP-SA is required to recognise Gram-positive bacteria and activate the Toll signalling pathway [112,113,114]. Although there are several studies on the role of this PRR in insects, the involvement in AMPs expression as an infection response of the homolog in *T. molitor*, Tm-PGR-SA (Genbank AB219970), was studied by Keshavarz et al. in 2020. Tissue gene expression analysis indicated that Tm-PGRP-SA was constitutively expressed in the late-instar fat body, hemocytes, and gut in the absence of microbial challenge, with a significant increase in the fat body and gut upon *E. coli*, *S. aureus*, and *C. albicans* infection. Furthermore, larval silencing increased the mortality rates for all microbial challenges assayed. TmPGRP-SA was also required to regulate the expression of eight AMPs genes and Tm-DorsalX2 in a microbe- and tissue-dependent manner, except for hemocytes. Overall, this experimental evidence established that the mortality observed in *T. molitor* infected *larvae* following TmPGRP-SA knockdown could be attributed to the compromised or suppressed TmPGRP-SA-mediated signal transduction required for the antimicrobial defence [115].

### 2.10. Scavenger Receptors (SRs)

SRs are a family of multifunctional proteins characterised by multi-domain structures. SRs are grouped into nine heterogeneous classes (SR-A–SR-I), and they are involved in removing cellular debris, oxidised low-density lipoproteins, and pathogens. The SR family represents an important part of the innate immune defence by acting as phagocytic receptors, especially in responses to bacteria. The SRs have been reported to recognise several different microbial structures, including lipoteichoic acid (LTA), lipopolysaccharide (LPS), bacterial CpG DNA, and yeast zymosan/β-glucan [116]. Among the nine SR classes, SR-C, a type I transmembrane glycoprotein, has been found only in certain invertebrates. In *D. melanogaster*, the SR-C has been implicated as a major phagocyte receptor in phagocytosis of gram-positive and -negative bacteria (Figure 1) [117].

The SR-C gene has also been identified in *T. molitor* (Tm-SR-C)*,* whose expression crucially influences antifungal and antibacterial immunity. Using quantitative PCR (qPCR), Gon Kim et al. studied the tissue-specific expression of Tm-SR-C after the injection of microorganisms, such as *E. coli*, *S. aureus*, and *C. albicans*. The SR-C is expressed constitutively in all tissues from late instar *larvae* to 2-day-old adults, with the highest transcript levels observed in hemocytes of *larvae* and adults. RNA silencing of Tm-SR-C transcripts strongly increased microbial susceptibility, revealing that the protein is required for the insect’s survival. Additionally, in TmSR-C-silenced *larvae*, there was a decline in the rate of microorganism phagocytosis, strongly influencing larval survivability against microbes. Overall, these findings indicate that TmSR-C plays a role in phagocytosing not only fungi but also gram-negative and -positive bacteria in *T. molitor* [118].

### 2.11. Immune Response Process

*T. molitor* triggers a potent endogenous immune response to combat infections and represents an excellent model for studying the biochemistry of immunity. Even though much research has been dedicated to understanding immune system regulation in adult and larval insects, recent studies demonstrated that insect eggs could induce an effective immune response to protect against pathogens [119]. However, immune gene expression was shown to be activated in insect eggs not only by a pathogen attack but also by transgenerational immune priming (TGIP). This mechanism refers to the transfer of the parental immunological experience to its progeny, preparing the eggs for the impending danger and triggering differential expression of immune-related genes [120].

Jacobs et al. investigated the endogenous immune response in eggs and adults of *T. molitor*, showing that many immune genes are induced in adults and eggs with comparable transcription levels [121]. After infection with *E. coli* and *Micrococcus luteus,* they measured gene expression of immune-related sequences associated with AMPs, pattern recognition proteins, toll receptors, and gram-negative binding proteins (GNBPs). Several AMPs were induced after infection, including Tm-Attacins A-D, Tm-Coleoptericin A-C, Tm-Thaumatin 1-3, and Tenecin 3. Attacins are known for their bactericidal activity against Gram-negative bacteria (see Supplementary Table S1 for further details) [122] and Coleoptericins for their activity against both Gram-negative and Gram-positive bacteria [123]. The high induction of these proteins in infected eggs may confer protection to the eggs against both Gram-negative and Gram-positive bacteria. Furthermore, clear induction of Tm-PGRP-SA and Tm-PGRP-SC2 in *T. molitor* eggs was recorded, while more modest increases were observed in adults. Even if these findings are in line with previous studies in *Tribolium castaneum* [124], the authors did not detect any change in GNBP expression in eggs and adults of *T. molitor* (Figure 1) [121].

Consistent with the results discussed above, *T. molitor* generates a remarkable immune response against bacteria. Moreover, *T. molitor* is also characterised by a long-lasting humoral immune response to control persistent infection and defend against chronic reinfection. *Staphylococcus aureus* infection in *T. molitor* is an attractive invertebrate model for studying persistent bacterial infection. Expressed sequence tags (ESTs) identified in *T. molitor* after infection with *S. aureus* via a suppressive-subtractive hybridisation (SSH) approach, Dobson et al. identified mRNAs coding for various oxidative enzymes and two antimicrobial peptides. The transcription of immune effectors, such as multicopper oxidase, tyrosine hydroxylase, and attacin, was significantly upregulated in infected *larvae*. Other immune effectors, such as ferritin and tenecin-1, were near-significantly modulated by the treatment. The effector pro-PO was the only one to be down-regulated by infection (see Supplementary Table S1 for further details) [125]. Overall, these data indicate a simultaneous expression of immune effectors as a hallmark of the *T. molitor* immune response.

An interesting study by Zanchi et al. assessed the effect of AMP knockdowns on the survival of the beetles and the survival of *S. aureus* [126]. Simultaneous knockdown of several AMPs, including Tenecin 1, the coleoptericin Tenecin 2, and the attacin Tenecin 4, results in increased mortality and elevated bacterial loads. Interestingly, the knockdown of only Tenecin 1 confers no survival benefit but reduces bacterial loads. Conversely, the single knockdown of the coleoptericin Tenecin 2 contributes to survival without affecting bacterial loads. These authors also found that attacin Tenecin 4 did not affect mortality (see Supplementary Table S1 for further details). Overall, these data indicate that reducing the expression of a few AMPs is sufficient to produce an effect on both bacterial presence and survival in the host.

## 3. Discussion

Although among insect models, *D. melanogaster* is one of the most studied for characterising Toll and IMD signalling pathways [127,128,129], its application as an alternative animal model poses several limitations. Indeed, understanding the biochemical mechanisms of these signalling pathways can be biased by numerous exogenous-dependent activating factors. Among these, the insect developmental stage and/or the occurrence of infections make a complete characterisation of the involved mechanisms difficult [112,130].

Furthermore, biochemical studies on immunity require a relatively large insect model to enable the collection of sufficient hemolymph samples, tissues for histochemical studies, and the presence of a phagocytosis system for host-pathogen interactions studies (Table 2).

Despite insects lacking an adaptive immune system, the innate humoral component shows many similarities with mammals. However, there are also significant differences in the pathways and mechanisms of each response. The NF-kb activation pathways are among the most important similarities found in both animals. In mammals, NF-κB is involved in the production of cytokines and co-stimulatory molecules, as well as cell survival and apoptosis through the activation of the Toll-like and TNF-α pathways [131].

On the other hand, in insects, the homologs of NF-κB (Dorsal and Relish) are activated by the Toll and IMD pathways. They are directly implicated in humoral innate immune responses through the transcription of AMPs. These transcription factors are responsible for insect and mammals’ resulting antimicrobial response [132]. Toll pathway activation in mammals occurs directly by binding an associated microbial component to their specific Toll receptor. In insects, the activation occurs indirectly when microbial invasion induces the production of a Spätzle protein that can bind to Toll receptors [133]. Following the activation of Toll by Spätzle, the adaptor protein MyD88 is recruited to the TIR domain of the Toll receptor, leading to the activation of the inhibitor protein Cactus. This process allows the release of Cactus from Dorsal if which are free to translocate into the nucleus, triggering AMP transcription [134].

A very similar process occurs in mammalian Toll-like signalling. Following the binding of microbial molecules, such as peptidoglycan or lipopolysaccharide, the Toll-like receptor MyD88, insect MyD88 homolog, is recruited to the TIR domain leading to the phosphorylation and degradation of I-κβ, Cactus homolog, thus initiating the translocation of NF-κB (Dorsal homolog), into the nucleus for the transcription of co-stimulatory molecules, cytokines, and chemokines [135]. The IMD pathway also has similarities with the mammalian tumour necrosis factor-α (TNF-α) and Toll-like pathways. In insects, the IMD pathway is initiated by binding peptidoglycan to PGRPs). In contrast, in mammals, the TNF-α pathway is initiated by the binding of TNF-α to tumour necrosis factor receptor 1 (TNFR1) [136,137].

These signalling pathways also regulate essential processes of the insect’s innate immune system, such as phagocytosis, degranulation, and coagulation, all mediated by hematocytes [138].

Insect hematocytes have similar functions to mammalian blood such as transport of nutrients, catabolites, and signal molecules; however, they have no role in oxygen transport [139]. For example, four different types of hemocytes involved in phagocytosis, nodulation, and encapsulation have been identified and characterized in the hemolymph of *G. mellonella* [140,141]. Furthermore, hemocytes from *G. mellonella* immunostimulated larvae express a protein with high homology to human calreticulin present in mammalian neutrophils, which is reported to be involved in the discrimination between self and non-self in cellular defence responses [142]. After phagocytosis, pathogens are removed by several mechanisms, including the secretion of lytic enzymes, (i.e., degranulation), and the production of reactive oxygen species generated by the oxidative burst and catalysed by the NADPH oxidase complex [143]. In *G. mellonella* hematocytes, human neutrophil’s homologous proteins essential for the generation of superoxide anions, namely the p47 and p67, have been identified. In human neutrophils, the functional NADPH complex is formed after the translocation of the p47 and p67 proteins from the cytosol to the plasma membrane [144].

Coagulation promotes hemostasis by inducing the formation of an insoluble matrix/clot in insect hemolymph and mammalian blood [132]. Similarities between insect and mammalian coagulation cascades can be observed in the family of transglutaminases that are involved in clot hardening. Insect transglutaminases are homologous to the human clotting factor XIIIa. In addition, similarities in protein sequence have also been observed between insect hemolectin and human von Willebrand factor, a glycoprotein involved in hemostasis [5].

The insect-specific humoral response is also characterized by insect-specific AMPs, metalloproteinase inhibitors (IMPIs), and proteins involved in the phenoloxidase pathway. Proteomics studies have recently shown that the molecular mechanisms regulating AMP production are regulated, in part, by non-coding microRNAs (miRNAs), as occurs in vertebrates [145]. Antimicrobial peptides play an important role in innate immunity by exhibiting broad-spectrum immunomodulatory and/or microbicidal activity [146,147]. Insect AMPs are secreted mainly from hemocytes, the fat body, the digestive tract, the reproductive tract, and salivary glands. In vertebrates, AMPs are produced by epithelial surfaces and phagocytic cells [148]

An insect metalloproteinase inhibitor IMPI-1 was isolated in the hemolymph of *G. mellonella*. IMPI-1 is a glycosylated and thermostable peptide with a molecular weight of 8.6 kDa, containing five intermolecular disulfide bonds that inhibit zinc-containing metalloproteinases [149]. On the other hand, Prophenoloxidase (PRO-po) is released from the oenocytoids and subsequently activated by a serine protease after infection recognition. This enzyme displays sequence regions similar to the C3 and C4 proteins of vertebrate complement [150].

Although there are many significant differences between the humoral component of the insect and mammalian immune systems, the similarities are sufficient to permit the usage of insects as models for studying host-pathogen interactions [131].

For this reason, in recent years, *T. molitor* has become a commonly used model for biochemical and molecular studies of innate immunity pathways and their components [21,40]. Due to these significant similarities, this review provided a comprehensive overview of the most recent knowledge on gene expression studies of *T. molitor* immunity in relation to microbial infection (Table S1).

In addition to the well-known cellular component responsible for pathogen recognition and humoral response liable for AMPs production [45], this review provided a comprehensive overview of the most recent knowledge on *T. molitor* immunity and host-pathogen interactions. Although the current information on genome sequencing (GenBank GCA_907166875.3) [42] has allowed the study of new factors associated with the immune response or the deepening of those already known, several aspects still remain open, namely the possible crosstalk between IMD and Toll pathways mediated by Tm-Reli [50] and Tm-DorX2 [60] along with the role of Tm-apoLip-III in the induction of AMPs expression [74].

As in vertebrates, also in insects stress response mediators are able to alter (i.e., reconfigure) the immune system [151]. Indeed, some intracellular stress response pathways converge on immune genes transcription signalling pathways. Lipid transport proteins (i.e., apoLip-III) are involved in both stress and immune responses, leading to reduced disease resistance when shifted to the stress response system [152]. In insects, the stress response induces a pro-inflammatory state that probably potentiates early immune responses, easing the loss of the molecular resource, such as lipid transport [153]. Therefore, intracellular responses to stress may also have an impact on disease resistance. Without the effects of stress hormones on immune function, resistance to disease would drop even more rapidly during and immediately after stress conditions [154,155]

## 4. Conclusions

The usage of Coleoptera as whole insects, their organs, cells, or bioactive molecules, along with their symbiotic microbes, in the fields of medicine, agriculture, and industry has increased significantly [156].

Yellow biotechnology’, named after the yellow colour of the insects’ hemolymph by Vilcinskas et al. in 2010 [157] has already yielded important results in medical, pharmaceutical, agricultural, and industrial applications [158]. Worthy examples are beetles AMPs or coleptericins, such as the ones discovered in *Zophobas atratus*, *T.Tribolium castaneum*, *H. axyridis, Acalolepta luxuriosa* [159] and Zophobas morio [160].

In this scenario, due to Coleoptera potentially high biomedical or industrial significance, it is essential to further investigate insect physiology and immune system mechanisms.

Therefore, further research is needed on *T. molitor* to produce more evidence to be well-accepted as an alternative insect model employed in the routine laboratory setting for host-pathogen interaction and immunity studies.

## Figures and Tables

**Table 1:** *T. molitor* lifecycle.

Stage	Lifetime
*Larva*	3 to 4 months
Pupa	10–20 days
Adult	1 to 3 months
Egg	10–12 days

**Table 2:** *Tenebrio molitor* advantages over other invertebrate models as in vivo model host.

	Invertebrate Model				Advantages
	*G. mellonella* [13]	*C. elegans* [39]	*D. melanogaster* [40]	*T. molitor*	
**Grown** **temperature**	37 °C	From 15 to 25 °C	From 16 to 29 °C	From 25 °C to 37 [19]	Suitable model for human pathogens study
**Life span**	Short	Long	Long	Long with several larval instars [21]	Long-term studies on different larval instars can be carried out
***Larvae* size (length)**	3 to 30 mm	1 mm	3 mm	10–28 mm [36,37]	Extraction of a considerable volume of hemolymph, furthermore microorganisms inoculum can be administered parentally into the hemolymph
**Tissue** **recovery**	Possible	Impossible	Impossible	Possible	Tissue studies can be performed
**Phagocytosis**	Present	Absent	Absent	Present	Information about host-pathogen interactions

## Data Availability

Not applicable.

## Supplementary Materials

The following supporting information can be downloaded at: https://www.mdpi.com/article/10.3390/microorganisms10101983/s1; Table S1: Reviewed studies’ main features.
