# Supplementary material for: Emerging Evidence on Tenebrio molitor Immunity: A Focus on Gene Expression Involved in Microbial Infection for Host-Pathogen Interaction Studies
Source: Microorganisms. 2022 Oct 7;10(10):1983. doi: 10.3390/microorganisms10101983 (PMC9611967; doi:10.3390/microorganisms10101983)
Supplement: Supplementary file 1 [file microorganisms-10-01983-s001.zip › microorganisms-1889716-supplementary.pdf]

Table S1. Reviewed studies main features. EG: egg YiL: young instar larvae; MiL: middle instar larvae; LiL: late instar larvae; PP: pre pupae; P1-P7: pupae day 1-7; A1-A5: adult day 1-5; IT: intertegument; HC: hematocytes; HL: hemolymph; GT: gut; MGT: midgut; EP: epidermidis; FB: fat body; MTS: Malpighian tubes; OV: ovary; TS: Testis; WB: whole body AMPs: antimicrobial peptides; cDNA FLC: cDNA Full-Length Cloning; RNAi MI: RNAi MI

T. molitor Gene/Protein (GenBank mRNA) (GenBank Protein*)	Product	Function	Developmental/ Tissue-Specific Expression	Microbial challenge Microorganism	Methods	Study main findings	First Author and pubblication year
			**NF-kB**				
**Relish (MK863367)**	NF-κB transcription factor	Immune-deficiency (Imd) pathway	EG, YiL; LiL,PP,P1–P7, A1-A5/ FB, MTs, GT, IT, HC, OV, TS	E. coli strain K12 S. aureus strain RN4220 C. albicans strain AUMC 13529	cDNA FLC in silico Analysis Expression analysis RNAi MI	Relish homologue, expressed in all immune tissues, with high expression level in the gut was identified	Keshavarz et al. 2020
**Relish (MK863367)**	NF-κB transcription factor	Immune-deficiency (Imd) pathway	YiL/ HC, FB, GT	Listeria monocytogenes strain ATCC 7644	Expression analysis RNAi MI AMPs and Autophagy genes expression Bioassay	Pivotal roles in regulation of AMP genes and induction of autophagy genes in response to L. monocytogenes	Keshavarz et al. 2020
**IKKγ (CAH1373157*)**	Regulatory subunit of the IκB kinase (IKK) complex	Regulation of NF-κB signaling pathway	EG YiL LIL, PP, P1–P7, A1–A5/ IT, GT, FB, MTs,HC	E. coli strain K12 S. aureus strain RN4220 C. albicans	in silico Analysis Expression analysis AMPs and NF-kB expression RNAi MI Bioassay	Important factor in the antimicrobial innate immune response	Ko et al. 2020

**IKbKε (MZ708789)**	Inhibitor of nuclear factor kappa-B kinase subunit epsilon	regulator of the NF-κB pathway against pathogenic invasion	EG, YiL, LiL, PP, P1–P7, A1–A5/ IT, HC, GT, FB, MTs, OV and TS	Escherichia. coli strain K12, Staphylococcus. aureus strain RN4220, Candida. albicans	cDNA FLC in silico Analysis Expression analysis RNAi MI Bioassay AMP and NF-κB expression	Important factor in antimicrobial innate immune responses	Ko et al. 2021
**MyD88 (HF935083)**	Myeloid differentiation factor 88	Adaptor protein involved in TLR signal processing	LiL PP, P1-P3-P5 A1-A3-A5/ IT, GT, FB, MTs, HC, OV and TS	E. coli strain K12 S. aureus strain RN4220	cDNA FLC in silico Analysis Expression analysis RNAi MI Bioassays	Gene silencing led to reduced host resistance to S. aureus infection	Patnaik t al. 2014
**Cactin (KY618833)**	Interactor of IκB factor	Embryonic polarity controller and regulator of the NF-κB signaling pathway	LiL, PP P1-P7, A1-A2/ IT, GT, FB, MTs, HC, OV and TS	E. coli strain K12 S. aureus strain RN4220 L. monocytogenes ATCC 7644 C. albicans	cDNA FLC in silico Analysis Expression analysis RNAi MI AMPs expression Bioassay	Important regulator of innate immunity, mediating AMP responses against both Gram-positive and Gram-negative bacteria	Jo et al. 2017
**TmDorX2 (MN056348)**	NF-κB transcription Factors family	Downstream component of the Toll pathway	EG, YiL, LIL, PP, P1-P7 A1-A5/ GT,HC,IT,MTs, FB, OV and TS	E. coli strain K12 S. aureus strain RN4220 C. albicans	cDNA FLC in silico Analysis Expression analysis RNAi MI AMPs expression	Important role in promoting the larval survival against bacterial and fungal infection	Keshavarz et. al 2019
**Immune deficiency death domain protein (IMD)**							

**IMD (MK121950)**	Immune deficiency death domain protein	IMD/NF-κB humoral and epithelial immune responses to Gram-negative bacteria and viruses	LiL, PP, P1-P7 A1-A2/ GT,HC,IT,MTs, FB, OV and TS	E. coli strain K12<br>S. aureus strain RN4220<br>C. albicans	cDNA FLC<br>in silico Analysis<br>Expression analysis<br>RNAi MI<br>AMPs expression	Required to confer humoral immunity against the E. coli by inducing the expression of AMPs.	Jo et al. 2019
**Damage-Associated Molecular Pattern (DAMP)**							
**Dsp1 (MW589636)**	High mobility group protein (Dorsal Switch Protein 1)	Immune responses by hemocyte nodule formation against infection	EG, YiL; LiL, P, A/ HL, FB,MGT, EP	Enterococcus mundtii	cDNA FLC<br>in silico Analysis<br>Expression analysis<br>RNAi MI<br>Nodulation assay<br>AMPs expression<br>PA2 and PO enzyme assay	Mediate immune responses upon immune challenge.	Mollah et al. 2021
**Apolipophorin III (apoLp-III)**							
**apoLp-II (HG316496)**	Apolipophorin III	Lipid transport and immune response	LiL/ Whole body	L. monocytogenes ATCC 7644<br>Escherichia coli P22	in silico Analysis<br>Expression analysis<br>Bioassays	Play an important role in innate immune responses against bacterial pathogens	Noh et al. 2014
**Immunity modulators**							
**14-3-3z (KP099938)**	Regulatory proteins	Phosphorylation-dependent signaling pathways	LIL, PP, P1-P7 A1-A2/ GT,HC,IT,MTs, FB, OV and TS	E. coli K12<br>C. albicans	cDNA FLC<br>in silico Analysis<br>Expression analysis<br>Peptide-Base Antiserum	Essential in the host defense mechanisms against bacteria and fungi.	Seong et al. 2018

					Subcellular localization RNAi MI Bioassay		
**14-3-3ε (KP099937)**	Phosphorylated serine-binding proteins	Signaling molecules in biological processes	Larvae/ FB, GT and HC	E. coli strain K12 S. aureus strain RN4220 C. albicans	cDNA Cloning and in Silico Analysis Expression analysis RNAi MI Antibacterial and Bioassay	Required to maintain innate immunity by enabling antimicrobial secretion into the hemolymph	Seo et al. 2016
**SOCS5 (MK292064) SOCS6 (MK292065) SOCS7 (MK292066)**	Cytokine signaling	Janus kinase (JAK)-signal transducers negative regulation and activators of transcription (STAT) pathway	EG, YiL, LIL, PP, P1-P7 A1-A5/ GT,HC,IT,MTs, FB, OV and TS	*E. coli* strain K12 *S. aureus* strain RN4220 *C. albicans*	cDNA Full-Length Cloning *in silico* Analysis Expression analysis Bioassay	Involvement of TmSOCS type-I subfamily in the host immune response	Patnaik et al. 2019
**Autophagy**							
**ATG3 (KF670693) ATG5 (KF670694)**	Autophagy-related genes	Autophagy process activator	LiL, P1–P7, A1–A2/ GT, HC, IT, MTs, FB, OV and TS	*L. monocytogenes* ATCC 7644	cDNA Full-Length Cloning *in silico* Analysis Expression analysis RNAi upon microbial infection Bioassays	Role in mediating autophagy-based clearance of *Listeria*	Tindwa et al. 2015
**ATG13 (KJ778621)**	Autophagy-related genes	Autophagy process activator	LiL, A2/ FB, GT,HC,IT, MTs, OV and TS	*E. coli* strain K12 *S. aureus* strain RN4220	cDNA Full-Length Cloning *in silico* Analysis Expression analysis	*T. molitor* gene homolog was identified and by	Lee et al. 2015

					RNAi upon microbial infection Bioassays	bioinformatics approach	
**ATG8 (KM676434)**	Autophagy-related genes	Autophagy process activator	LiL, P1-P7, A1-A/ GT, HC, IT, MTs, FB, OV and TS	*L. monocytogenes* ATCC 7644	cDNA Full-Length Cloning *in silico* Analysis Expression analysis RNAi upon microbial infection bioassay studies *E. coli* expression Immunofluorescence staining and confocal microscopic analysis	Play a role in mediating autophagy-based clearance of *Listeria monocitogenes*	Tindwa et al. 2015
**ATG6 (MN259540)**	Autophagy-related gene-6	Autophagosome formation and autolysosome maturation	EG, YiL, LIL, PP, P1-P7 A1-A5/ GT,HC,IT,MTs, FB, OV and TS	*E. coli* strain K12 *S. aureus* strain RN4220 *L. monocytogenes* *C. albicans*	cDNA Full-Length Cloning *in silico* Analysis Expression analysis RNAi upon microbial infection Bioassay	Essential role in anti-microbial defense against intracellular bacteria.	Edosa et al. 2020
**Toll receptors**							
**Toll-7 (MK234903)**	Toll receptor	Antiviral autophagy	LIL, PP, P1-P7 A1-A2/ GT,HC,IT,MTs, FB, OV and TS	E. coli strain K12 S. aureus strain RN4220 C. albicans	cDNA FLC in silico Analysis Expression analysis RNAi MI AMPs expression	Important role in regulating the immune response to E. coli.	Park et al. 2019
**Spätzle**							
**Spz6 (CAH1365756*)**	Dimeric cytokine ligand	Regulation of AMP production in response to infection.	EG, YiL, LIL, PP, P1-P7 A1-A5/	E. coli strain K12 S. aureus strain RN4220	in silico Analysis Expression analysis RNAi MI	Regulates AMP expression and increases the survival	Edosa et al. 2020

			GT,HC,IT,MTs, FB, OV and TS		AMPs expression	against E. coli and S. aureus.	
**Spz3 (CAH1376064*) Spz4 (MT075617) Spz6 (CAH1365756*) Spz-like (MZ708792)**	Dimeric cytokine ligand	Critical role in the regulation of AMP production in response to infection.	YiL/ HC,FB,GT	E. coli strain K12 S. aureus strain RN4220 C. albicans	AMPs Activity Study Expression analysis RNAi upon biosurfactants treatment Bioassay	Biosurfactants induce the AMPs via spätzle genes, increasing the survivability E. coli.	Edosa et al. 2020
**Spz1b (MZ708791)**	Dimeric cytokine ligand	Toll pathway activation and AMP production upon pathogen challenge	YiL, PP, P1-P7, A1-A5 LiL/ IT, FB, HC, GT MTs, OV and TE	E. coli strain K12 S. aureus strain RN4220 C. albicans strain AUMC 13529	cDNA FLC in silico Analysis Expression analysis RNAi MI Bioassay AMPs expression	Gram-negative sequestration by AMPs regulatory action	Bae et al.2021
**Spz5 (MW916536)**	Dimeric cytokine ligand	Toll pathway activation and AMP production upon pathogen challenge	EG, YiL, LiL, PP, P1–P7, A1–A5/ IT, GT, FB, MTs; OV and TS	E. coli strain K12 S. aureus strain RN4220 C. albicans strain AUMC 13529	cDNA FLC in silico Analysis Expression analysis RNAi MI NF-κB expression	Defined role in innate immunity by regulating AMP expression in MTs in response to E. coli.	Kojouret al. 2021
**Spz-like (MZ708792)**	Dimeric cytokine ligand	AMP production regulation.	EG, YiL, LiL, PP, P1-P7, A1, A5/ IT, HC, GT, FB, MTs OV and TS	E. coli strain K12 S. aureus strain RN4220 C. albicans strain AUMC 13529	cDNA FLC in silico Analysis Expression analyisis RNAi MI Bioassay AMPs and NF-kB expression	Critical role in the regulation of AMP production due to E. coli infection.	Jang et al. 2021

**Peptidoglycan recognition proteins**							
**PGRP-LE (HF935084)**	Peptidoglycan recognition protein	DAP-type peptidoglycan selective binding, IMD and proPO pathways activation, autophagy induction	LiL, PP, P1–P7, A1-A2/ whole body	L. monocytogenes ATCC 7644 E. coli ATCC 25922	cDNA FLC in silico Analysis Expression analysis RNAi MI Bioassay	Defense protein necessary for survival against L. monocytogenes. infection	Tindwa et al. 2013
**PGRP-LE (HF935084)**	Peptidoglycan recognition proteins	NF-κB transcription proteins translocation and AMPs expression	Larvae and adults/ IT, FB, HC, GT, MTs, OV and TS	E. coli strain K12 S. aureus strain RN4220 C. albicans	Expression analysis AMPs and NF-kB expression RNAi MI Bioassay	Important gut microbial sensor that induces AMPs	Keshavarz et al. 2020
**GNBP3 (AB10884)**	Gram-negative-binding protein 3	Prophenoloxidase (proPO) cascade activator and Toll signaling pathway inducer	Whole body larvae	Beauveria bassiana JEF-007	Expression analysis RNAi MI	Essential to induce downstream Tenecin 1 expression against B. bassiana JEF-007.	Yang et al. 2018
**PGRP-SA (AB219970)**	Peptidoglycan recognition protein-SA	Recognition of non-self molecules.	EG, YiL, LiL P1-P7 A1-A5/ IT, FB, HC, GT, MTs, OV and TS	E. coli strain K12 S. aureus strain RN4220 C. albicans strain AUMC 13529	cDNA FLC in silico Analysis Expression analysis RNAi MI AMPs expression Bioassay	Multivalent binding properties to bacteria and fungi as a positive regulator in the expression of eight of	Keshavarz et al. 2020

						fourteen AMP genes	
**Scavenger receptors**							
**SR-C (KY977453)**	scavenger receptor class C	Cellular debris, oxidized low-density lipoproteins, and pathogens remover	LiL, A1-A2/ GT,HC,IT,MTs, FB, OV and TS	C. albicans	cDNA FLC in silico Analysis Expression analysis RNAi MI Peptide-specific antibody Phagocytosis assay	Pivotal role in gram-negative and -positive bacteria phagocytosing	Kim et al. 2017
**Immune response process**							
**Attacin-1a (MF754109) Attacin-1b (MF754110) Attacin-C ((MF754108) Cecropin2 (MT905403) Coleoptericin-A (KF957599) Coleoptericin-B (KF957600) Coleoptericin-C (KF957601) Thaumatin-1 (CAH1373168*) Thaumatin-2 (KAH0809363*) Tenecin3 (U21482)**	AMPs, GNBP, toll receptor and pattern recognition receptors	Endogenous immune responders	Whole beetles body and eggs	E. coli Micrococcus luteus	Expression analysis Bioassay	The eggs are capable of an impressive endogenous immune response	Jacobs et al. 2017

**Putative-defense-protein-hdd11 (KAH0812851*) GNBP (AB363981) Toll (MK234903) PGRP-SA (AB219970) PGRP-SC2 (AB560751)**							
**Multicopper oxidase (CAH1375319*) Tyrosine hydroxylase (MN520434) Ferritin (CAH1370634*) Attacin 2 (MF754109) Tenecin 1 (D17670) ProPO (AB020738)**	Enzymatic cascades associated with melanin production Iron scavengers AMPs Prophenoloxidase	Immune effectors	LiL/ Whole body	S. aureus SH1000 tetracycline-resistant JLA513	cDNA FLC in silico Analysis Expression analysis	With the exception of ferritin, the broad up-regulation of the target immune effectors in infected larvae suggests are up-regulated in response to S. aureus infection	Dobson et al. 2012
**Tenecin 1 (D17670) Tenecin 3 (U21482) Tenecin 4 (AB669089)**	AMPs	Immune effectors	Whole body beetles/ HL	Staphylococcus aureus SH 1000 containing a Tetracyclin-resistance cassette	RNAi MI Bioassay	The expression of multiple AMPs increases survival and contributes to infections control and tolerance	Zanchi et al. 2017
